# Increased aqueous flare after intravitreal brolucizumab injections compared to aflibercept in neovascular age-related macular degeneration

**DOI:** 10.1007/s00417-025-07047-6

**Published:** 2025-11-29

**Authors:** Yuto Hashimoto, Yusuke Arai, Hidenori Takahashi, Hironobu Tampo, Rika Kondo, Hironori Takahashi, Hana Yoshida, Ryota Takahashi, Satoru Inoda, Toshikatsu Kaburaki, Yasuo Yanagi

**Affiliations:** 1 Department of Ophthalmology, Jichi Medical University, 3311-1 Yakushiji, Shimotsuke-shi, 329-0498 Tochigi Japan; 2 Center for Cyber Medicine Research, University of Tsukuba, 1-1-1 Tennodai, Tsukuba-shi, 305-8575 Ibaraki Japan; 3 Department of Ophthalmology and Micro-Technology, Yokohama City University Medical Center, Yokohama, Japan; 4 Retina Research Group, Singapore Eye Research Institute, Singapore, Singapore

**Keywords:** Aqueous flare, Anti-VEGF therapy, Neovascular age-related degeneration, Brolucizumab, Aflibercept

## Abstract

**Purpose:**

To evaluate aqueous flare values in patients with neovascular age-related macular degeneration (nAMD) receiving anti-vascular endothelial growth factor (VEGF) therapy, including brolucizumab.

**Methods:**

This retrospective study included 101 patients treated with intravitreal anti-VEGF injections at Jichi Medical University Hospital from March to July 2021. Aqueous flare values were measured in both affected and fellow eyes. The number of treated eyes was 28 for aflibercept and 73 for brolucizumab. Flare values were compared between affected and fellow eyes, and associations with age, gender, drug type, number of injections, disease duration, and time since last injection were analyzed. We also measured flare values from pre-filled syringes.

**Results:**

In unilateral treatment cases, brolucizumab-treated eyes had significantly higher aqueous flare values than fellow eyes (6.7 vs. 6.2 photon counts/ms, *P* = 0.0032), while no significant difference was observed with aflibercept (6.9 vs. 6.7 pc/ms, *P* = 0.55). Flare values increased significantly with age in the brolucizumab group (*P* = 0.0076) but not in the aflibercept group (*P* = 0.56). The number of brolucizumab injections did not alter flare values. Multivariate analysis identified age as the only significant factor associated with increased aqueous flare. The mean (standard deviation) flare values measured from pre-filled syringes were 430 (15.6) pc/ms for Beovu^®^ (brolucizumab) and 161.8 (31) pc/ms for Eylea^®^ (aflibercept).

**Conclusion:**

In nAMD, aqueous flare values were higher in brolucizumab-treated eyes and increased with age but were unaffected by the number of injections. Different flare values of each drug might affect the aqueous flare values within hours after injection.

## Key messages

*****What is known***:**

- Aqueous flare is widely used as an indicator of intraocular inflammation.

- Brolucizumab has received renewed attention because of its association with intraocular inflammation (IOI).

*****What is new***:**

- Eyes treated with brolucizumab showed higher aqueous flare compared to fellow eyes, whereas aflibercept-treated eyes did not.
- The high molar concentration of brolucizumab results in an intrinsic flare value of approximately 430 pc/ms in solution, which is estimated to transiently increase anterior chamber flare by about 0.3 pc/ms immediately after injection.

## Introduction

Brolucizumab is a humanized single-chain variable fragment (scFv) of an anti- vascular endothelial growth factor (VEGF) antibody [1]. Its high solubility allows for intravitreal administration at elevated concentrations. Combined with its small molecular size, which enhances tissue penetration and bioavailability, brolucizumab demonstrates improved durability and efficacy in the treatment of neovascular age-related macular degeneration (nAMD) compared with currently available therapies [2–4].

However, in the phase III HAWK and HARRIER studies, brolucizumab was associated with higher rates of aseptic intraocular inflammation (IOI), retinal vasculitis, and retinal vascular occlusion compared with aflibercept [4]. Since its market approval, the incidence of IOI with brolucizumab has remained notably higher than with other anti-VEGF agents. The spectrum of IOI associated with brolucizumab ranges from peripheral retinal vasculitis to occlusion of major retinal arteries near the optic disc or macula, potentially resulting in severe vision loss [5].

The underlying mechanisms of IOI following brolucizumab administration remain incompletely understood, highlighting the need for further investigation. While previous studies have evaluated aqueous inflammation associated with existing anti-VEGF therapies [6–8] there are limited reports characterizing the course of aqueous inflammation specifically following brolucizumab injections.

In this study, we report difference in aqueous flare values following intravitreal injections of aflibercept and brolucizumab.

## Methods

### Study design and ethical approval

This retrospective observational study was approved by the Institutional Review Board of Jichi Medical University (CU19-017) and conducted in accordance with the tenets of the Declaration of Helsinki. De-identified clinical data were retrospectively analyzed. Given the nature of the study, the Institutional Review Board granted an opt-out option and waived the requirement for written informed consent.

### Participants

A total of 101 patients with neovascular age-related macular degeneration (nAMD) who presented to Jichi Medical University Hospital between March and July 2021 were included. The study cohort consisted of 76 male and 25 female patients. Anti-VEGF agents administered were aflibercept in 28 eyes and brolucizumab in 73 eyes. The mean age of participants was 73.0 years (standard deviation [SD]: 7.0; range: 57–89 years).

### Procedure of aqueous flare measurement

At each visit, both eyes of all patients visiting our institution for neovascular age-related macular degeneration were routinely dilated, and when aqueous flare measurements were performed, both eyes were examined, including the fellow eye. A single, experienced ophthalmic photographer with a coefficient of variation (CV) of approximately 20%, considered highly reliable for this measurement, performed all laser flare meter assessments. An orthoptist initially participated in measurements but was later excluded due to unacceptable measurement variability. Aqueous flare measurements were performed on an opportunistic basis as part of routine ophthalmic imaging duties, depending on the availability of a single qualified technician who also handled other procedures. The timing of each measurement was not predetermined or aligned with treatment schedules, and the similar mean interval since the last injection observed between groups occurred coincidentally rather than by design. After pharmacologic mydriasis, aqueous flare values were measured in a dark room using a laser flare meter (FM-600; Kowa Corporation, Nagoya, Japan), which measures flare values only. Each eye was measured five times, and the mean value was used for analysis.

### Diagnosis of nAMD

All patients underwent fluorescein angiography for the diagnosis of nAMD diagnosis of nAMD and for classification into macular neovascularization (MNV) types 1, 2, and 3, except in cases where contraindications such as drug hypersensitivity, hepatic dysfunction, or recent cerebrovascular events were present. Indocyanine green angiography was also performed to identify polypoidal choroidal vasculopathy (PCV). Swept-source optical coherence tomography (OCT) was performed using either a Silverstone system (Nikon Corporation, Tokyo, Japan) or a DRI OCT Triton (Topcon Corporation, Tokyo, Japan), and spectral-domain OCT was conducted using an RS-3000 Advance (Nidek Co., Ltd., Aichi, Japan) at every visit.

### Data inclusion criteria

Medical records were reviewed up to three months prior to aqueous flare measurements. Eyes for which the most recent injection was aflibercept were assigned to the aflibercept group, and those for which it was brolucizumab were assigned to the brolucizumab group. All eyes were managed using a treat-and-extend (TAE) regimen. The switching of anti-VEGF agents was performed either when further extension of the treatment interval was not possible and a new drug with potentially longer durability became available, or when the disease failed to remain dry after switching. Therefore, the flare values measured just before switching were not considered to be particularly influenced by AMD disease activity.

### Data exclusion criteria

Exclusion criteria included eyes with a history of any ocular surgery (including intraocular or extraocular procedures) within three months of aqueous flare measurement, eyes under topical glaucoma medication, eyes with a history of sub-Tenon capsule or intravitreal steroid injections, and any other retinal disease or active nAMD in the fellow eye. In cases with multiple flare measurements, only the most recent measurement was included in the analysis.

### Comparison of aqueous flare values between treated and fellow eyes

Aqueous flare values were compared between the treated and fellow eyes within each group of eyes with neovascular age-related macular degeneration (nAMD) that had recently received aflibercept or brolucizumab injections.

### Difference in aqueous flare values with age

Linear regression analyses were performed between aqueous flare values in the treated eyes and patient age separately for the aflibercept and brolucizumab groups. Subsequently, both groups were combined, and regression lines were fitted between flare values and age for the treated and fellow eyes, respectively.

### Comparison in aqueous flare values by the number of previous injections

Linear regression analyses were performed between aqueous flare values and the total number of previous intravitreal injections. Scatter plots with fitted regression lines were generated to illustrate these relationships.

### Statistical analysis

Statistical analyses were conducted using JMP Pro software (version 18.02, SAS Institute Inc.). Continuous variables including age, sex, disease duration, number of anti-VEGF injections, and last injection interval were compared using unpaired t-tests. Within each anti-VEGF group, aqueous flare values in treated and fellow eyes were compared using paired t-tests, and aqueous flare values between the aflibercept and brolucizumab groups were compared using unpaired t-tests. To investigate difference in aqueous flare values with age, linear regression slopes of aqueous flare values plotted against age were compared using t-tests. Additionally, to compare in aqueous flare values by the number of previous injections, *t*-tests were performed on the slopes of aqueous flare values plotted against treatment duration and number of injections. Two-tailed *t*-tests were used throughout, with a significance threshold set at *P* < 0.05. As this was an exploratory study, no correction for multiple comparisons was applied.

### Measurement of flare values in Anti-VEGF drug syringes

Flare values were also measured in pre-filled syringes of brolucizumab (Beovu^®^, Novartis Pharmaceuticals Canada Inc.), aflibercept (Eylea^®^, Regeneron Pharmaceuticals, Tarrytown, NY, USA), and ranibizumab (Lucentis^®^, Genentech, South San Francisco, CA, USA) using the same laser flare meter. Each agent’s flare was measured directly through the pre-filled syringe rather than from aqueous samples. Five syringes of each formulation were assessed, and the mean and standard deviation were calculated.

This measurement was conducted to examine whether anti-VEGF agents with higher molar concentrations exhibit higher intrinsic flare values, as this could partially explain the greater aqueous flare sometimes observed after Beovu^®^ injections. Lucentis^®^ was also included, as no previous reports have described its flare characteristics, and its measurement was considered of academic interest.

## Results

### Patient characteristics

The baseline characteristics of the study participants are summarized in Table 1. A total of 28 eyes were included in the aflibercept group and 73 eyes in the brolucizumab group. The mean age was 72 years in the aflibercept group and 74 years in the brolucizumab group. The proportion of male patients was 70% and 75%, respectively. The mean total number of anti-VEGF injections received was 14 in both groups, and the mean duration since the last injection was 7.6 weeks in both groups. The total injection count included all types of anti-VEGF agents administered. Among the brolucizumab group, 41 eyes had received brolucizumab as the initial and continuous treatment, while the remaining cases had switched from other anti-VEGF agents but had undergone multiple brolucizumab injections before flare measurement. Patient background characteristics were generally well-balanced between the groups (Table 1).

**Table 1:** Characteristics of the participants

	Aflibercept (*n* = 28 eyes)	Brolucizumab (*n* = 73 eyes)	*P*-value(t-test) Aflibercept vs. Brolucizumab
Age (years, mean (SD))	72 (8)	74 (7)	0.45
Male (n (%))	19 (70)	48 (75)	0.65(Pearson’s chi-square test)
Duration of therapy (months, mean (SD))	42 (35)	33 (33)	0.26
Number of injections (mean (SD))	14 (9)	14 (12)	0.83
Last injection interval (weeks, mean (SD))	7.6 (2.3)	7.6 (2.4)	0.99

All values are expressed per eye. *SD *standard deviation

### Comparison of aqueous flare values between treated and fellow eyes

**Fig. 1:**
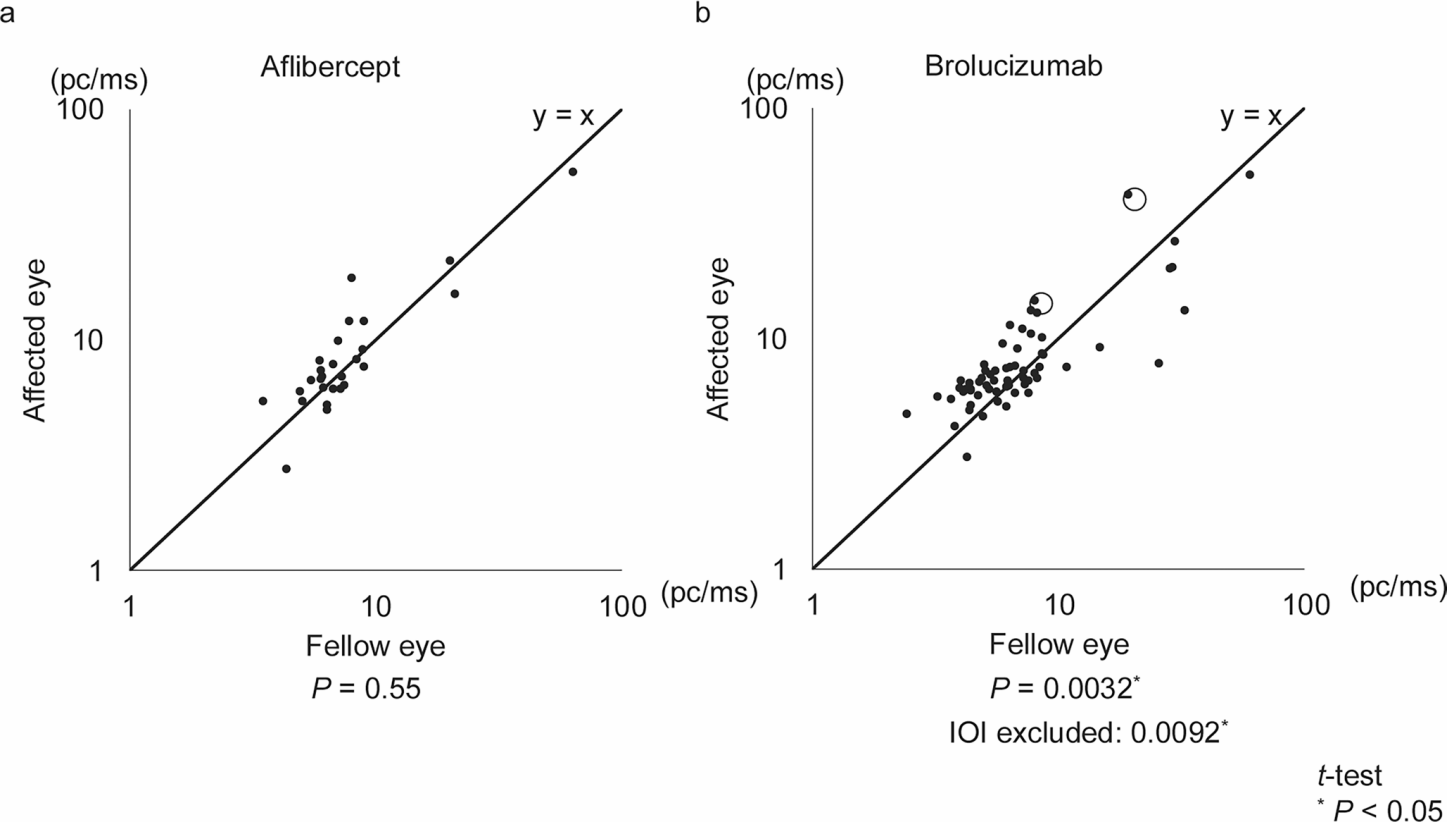
Comparison of aqueous flare values between the affected eye and the fellow eye in aflibercept and brolucizumab groups. A scatter plot showing aqueous flare values of the affected eye (vertical axis) and the fellow eye (horizontal axis). The diagonal line (labeled “y = x”) represents equal flare values between the affected and fellow eyes. In the brolucizumab group, flare values were significantly higher in the affected eyes compared to the fellow eyes. Data points corresponding to cases at the onset of intraocular inflammation (IOI) are circled. The results remained consistent after excluding IOI cases

When comparing aqueous flare values between the treated and fellow eyes within each group, aqueous flare values were significantly higher in the treated eyes of the brolucizumab group (6.7 [6.0–9.0] photon counts per millisecond (pc/ms), median [interquartile range; IQR]) compared to their fellow eyes (6.2 [4.7–8.0] pc/ms; *P* = 0.0032). This result remained consistent even after excluding cases of intraocular inflammation (IOI) (*P* = 0.0092) and in the combined analysis of both treatment groups (6.8 [6.0–9.0] vs. 6.3 [4.8–8.1], *P* = 0.010) (Table 2; Fig. 1).

**Table 2:** Comparison of aqueous flare values between the affected eye and the fellow eye in Aflibercept and Brolucizumab groups

	Aflibercept (*n* = 28 eyes)	Brolucizumab (*n* = 73 eyes)	*P*-value (unpaired t-test) Aflibercept vs. Brolucizumab
Affected eye flare (pc/ms, median (IQR))	6.9 (6.1—9.9)	6.7 (6.0—9.0)	0.64
Fellow eye flare (pc/ms, median (IQR))	6.7 (5.9—8.3)	6.2 (4.7—8.0)	0.5
nAMD (inactive) in fellow eye (n (%))	7 (25)	8 (11)	0.11
*P*-value (paired *t*-test) Affected eye vs. Fellow eye	0.55	0.0032*	

*IQR *interquartile range, *nAMD *neovascular age-related macular degeneration

* *P* < 0.05

### Difference in aqueous flare values with age

**Fig. 2:**
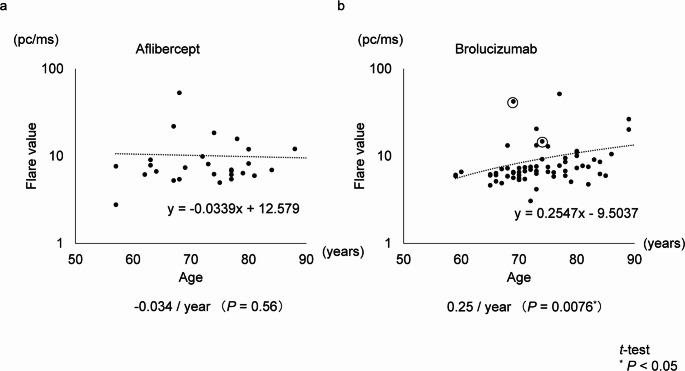
Association between aqueous flare values and age in the aflibercept and brolucizumab groups. This figure illustrates the relationship between aqueous flare values and patient age in the aflibercept and brolucizumab groups. In the brolucizumab group, aqueous flare values significantly increased with age at a rate of 0.25 pc/ms per year (*P* = 0.0076 < 0.05). In contrast, no significant association was observed in the aflibercept group (–0.034 pc/ms per year, *P* = 0.56). Data points corresponding to cases at the onset of IOI are circled

When assessing the relationship between aqueous flare values and age within each treatment group, aqueous flare values increased significantly with age in the brolucizumab group (0.25 pc/ms/year; *P* = 0.0076). No significant age-related difference in aqueous flare values was observed in the aflibercept group (−0.034 pc/ms/year; *P* = 0.56). These results were no difference when IOI cases were excluded (Fig. 2).

**Fig. 3:**
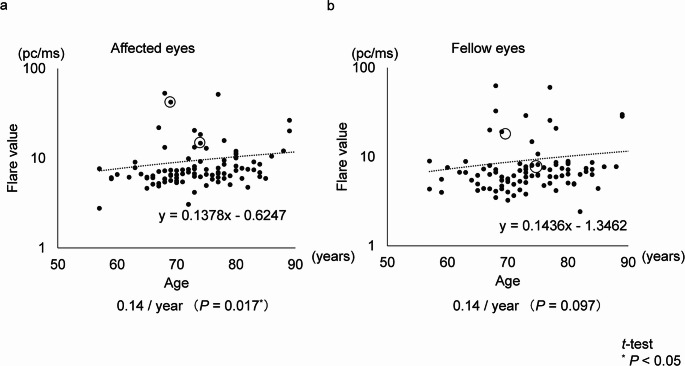
Difference in aqueous flare values with age in affected and fellow eyes. Difference in aqueous flare values with age in affected and fellow eyes. This figure depicts the progression of aqueous flare values with age in affected and fellow eyes across both treatment groups. In affected eyes, aqueous flare values significantly increased with age (0.14 pc/ms per year, *P* = 0.017 < 0.05). No significant age-related difference was observed in fellow eyes (0.14 pc/ms per year, *P* = 0.097). When analyzing all eyes combined, aqueous flare values significantly increased with age (0.14 pc/ms per year, *P* = 0.0051 < 0.05). Data points corresponding to cases at the onset of IOI are circled

When analyzing both groups combined, aqueous flare values in the treated eyes increased significantly with age (0.14 pc/ms/year; *P* = 0.017), whereas no significant difference was observed in the fellow eyes (0.14 pc/ms/year; *P* = 0.097). When both treated and fellow eyes were analyzed together, a significant age-related increase in aqueous flare values was detected (0.14 pc/ms/year; *P* = 0.0051) (Fig. 3).

### Comparison in aqueous flare values by the number of previous injections

**Fig. 4:**
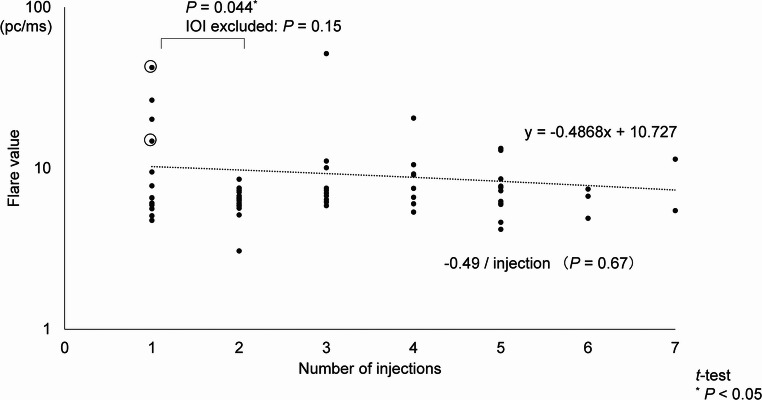
Difference in aqueous flare values with the number of brolucizumab injections. This figure shows differences in aqueous flare values associated with the number of intravitreal brolucizumab injections. No significant overall difference in aqueous flare values was observed with the number of injections (*P* = 0.67). Among 73 cases, 41 had switched from aflibercept. A significant increase in aqueous flare values was noted between the first and second injections (*P* = 0.044), although this difference was not significant after excluding IOI cases (*P* = 0.15). Data points corresponding to cases at the onset of IOI are circled

In the brolucizumab group, no significant difference in aqueous flare values was observed with the number of previous injections (−0.49 pc/ms/injection; *P* = 0.67) (Fig. 4). Among the 73 cases in the brolucizumab group, 41 had switched from aflibercept.

### Multivariate analysis

A multivariate analysis was conducted to assess the association between aqueous flare values in the treated eyes and factors including age, sex, type of anti-VEGF agent, duration of disease, number of injections, and weeks since the last injection (Table 3). Stepwise variable selection identified age as the only significant factor (*P* = 0.019). No multicollinearity was observed among explanatory variables, with variance inflation factors (VIFs) for all variables below 10.

**Table 3:** Multivariate analysis on factors related to the aqueous flare value in affected eyes

	Estimated value	*P*-value
Age	0.0081	0.019*
Sex (female)	−0.011	0.7
Type of drug (Aflibercept)	0.016	0.53
Duration of therapy	0.00028	0.78
Number of injections	−0.00032	0.92
Last injection interval	−0.011	0.31

* *P* < 0.05

### Flare values in Anti-VEGF syringes

The mean (SD) flare values measured from pre-filled syringes were 430 (15.6) pc/ms for Beovu^®^ (brolucizumab) and 161.8 (31) pc/ms for Eylea^®^ (aflibercept), and 57.9 (5.9) pc/ms for Lucentis^®^ (ranibizumab).

## Discussion

In this study, excluding cases of IOI, brolucizumab-treated eyes within the past three months showed higher aqueous flare than fellow eyes. Previous reports have shown that with bevacizumab, aqueous flare values remained unchanged on the day after intravitreal injection, decreased after one week, and showed a significant reduction after one month [9]. Conversely, one study reported a statistically significant increase in aqueous flare values only with bevacizumab; however, this difference was not considered clinically meaningful, and the authors concluded that bevacizumab does not induce intraocular inflammation [10].

In our cohort of nAMD, relatively high baseline flare values might have attenuated the apparent post-injection increase, reducing the likelihood of detecting a statistically significant change, as suggested in a previous study on diabetic macular edema [11]. Moreover, intraocular inflammation associated with brolucizumab is considered to be mediated by adaptive immune responses, which are typically enhanced by repeated exposure [19]. However, eyes with highly active nAMD and residual intraretinal fluid have been reported to show elevated aqueous flare values [20

In clinical practice, an increase in flare values of approximately 10 pc/ms is often considered clinically meaningful [12], and the median values observed in this study were well below this threshold. These variations in aqueous flare are thought to reflect the drug’s effect on suppressing neovascular activity in the posterior segment, which indirectly influences anterior chamber protein concentrations.

In contrast, the present study demonstrated that brolucizumab led to an increase in aqueous flare values, suggesting enhanced permeability of the blood-aqueous barrier. This may not necessarily reflect inflammation but rather indicate an alternative mechanism of increased barrier permeability. Previous reports have noted transient prolongation of the implicit time in flicker electroretinograms following intravitreal brolucizumab injections, presumed to result from alterations in retinal circulation due to its potent VEGF-suppressive effects [13]. We further hypothesized that high concentrations of brolucizumab in the anterior chamber, even in the absence of inflammation, might contribute to elevated aqueous flare values.

To evaluate the direct contribution of the anti-VEGF agents themselves to aqueous flare values, we measured flare values from pre-filled syringes of Beovu^®^ (brolucizumab), Eylea^®^ (aflibercept), and Lucentis^®^ (ranibizumab) using a laser flare meter. The mean flare value of Beovu^®^ was notably higher than those of Eylea^®^ and Lucentis^®^. According to the FDA’s product quality review for Beovu^®^, the absorption coefficient of brolucizumab at 280 nm is 2.209 × 10³ mL/(g·cm) [14]. In comparison, Veurink et al. reported the absorption coefficient for Lucentis^®^ to be 1.8 cm·mL/mg, with no significant difference [15]. The absorption coefficient, when combined with solution concentration and optical path length, encompasses absorption, scattering, and reflection effects.

The half-life of brolucizumab is approximately 3.0 [16]—9.0 [17] days, with levels decreasing to 0.16% after four weeks, as reported by Nimz et al. [16]. Given the dilutional effect within the vitreous and anterior chamber, the concentration of brolucizumab in the anterior chamber is typically about 1/40th of that in the vitreous. As such, the theoretical increase in aqueous flare values due to brolucizumab itself after four weeks is estimated at approximately 0.00017 pc/ms, a negligible value unlikely to influence clinical measurements. However, it is worth noting that about 1/1000 of the injected drug concentration can be detected in the anterior chamber within hours after injection, potentially increasing aqueous flare values by approximately 0.3 pc/ms in the case of Beovu^®^.

Additionally, prior studies have demonstrated age-related increases in blood-aqueous barrier permeability, leading to elevated protein concentrations and higher flare values in the anterior chamber [18]. Our findings are consistent with these observations, as aqueous flare values increased significantly with age. Multivariate analysis revealed age as the only significant predictor of aqueous flare values, while treatment duration, number of injections, and post-injection intervals were not associated factors.

Variance inflation factors (VIFs) were assessed to detect multicollinearity among variables in the regression analysis. VIF values exceeding 10 indicate strong multicollinearity, whereas values between 5 and 10 suggest moderate correlation. In this study, the VIF for age and other predictors remained below 10, suggesting low multicollinearity and confirming the independent effect of age on aqueous flare values.

A prior report indicated that patients with a history of more than 14 intravitreal injections and aqueous flare values exceeding 15 pc/ms may be at increased risk for IOI following brolucizumab injection [19]. In our study, although several cases exhibited aqueous flare values higher than those typically observed at the onset of IOI, none demonstrated clinical findings indicative of inflammation. No factors directly associated with increased aqueous flare values were identified. Consequently, it remains difficult to predict IOI based solely on laser flare meter readings. In cases where elevated aqueous flare values are detected, confirmation of IOI signs such as anterior chamber cells or vitreous haze should precede clinical decision-making.

This study has several limitations. First, the study design did not allow for paired, pre- and post-injection measurements in the same eye; instead, fellow eyes served as controls, and disease status may have varied between eyes. Second, as a single-center investigation, selection bias cannot be excluded. Third, the imbalance in sample size may have reduced the statistical power to detect significant differences in the aflibercept group. Fourth, pre-treatment flare data and standardized post-injection timing were not available because the flare photometer was only temporarily available at our institution, limiting the comparability of our results with previous studies. Finally, the study cohort was composed almost entirely of Japanese patients, limiting the generalizability of these findings to other ethnic populations.

In conclusion, nAMD eyes brolucizumab injection for less than 3 months had significantly higher aqueous flare values compared to fellow eyes. Aqueous flare values increased with aging. Neither aflibercept nor brolucizumab showed any difference in aqueous flare values with continued injection with TAE.

## Data Availability

The datasets generated during and/or analysed during the current study are available in the figshare repository, DOI: 10.6084/m9.figshare.29410361.

## Declarations

### Language editing

The authors used ChatGPT-4o (OpenAI, San Francisco, CA, USA) to assist with language editing on June 19, 2025, during the preparation of this manuscript.
